# Development of necrotizing enterocolitis in full-term infants with duct dependent congenital heart disease

**DOI:** 10.1186/s12887-022-03186-5

**Published:** 2022-04-02

**Authors:** Gwang-Jun Choi, Jinyoung Song, Hanna Kim, June Huh, I-Seok Kang, Yun Sil Chang, Se In Sung, Myung Chul Hyun

**Affiliations:** 1 Department of Pediatrics, School of Medicine, Kyungpook National University, Daegu, South Korea; 2 Department of Pediatrics, Samsung Medical Center, Sungkyunkwan University School of Medicine, Seoul, South Korea

**Keywords:** Congenital heart disease, Necrotizing enterocolitis, Patent ductus arteriosus

## Abstract

**Background:**

Although many studies have described an increased risk of necrotizing enterocolitis in duct dependent congenital heart diseases, very few have investigated its occurrence in full-term infants with duct dependent congenital heart diseases.

**Methods:**

To evaluate the characteristics and risk factors of necrotizing enterocolitis, we performed a retrospective review of 355 full-term infants with duct dependent congenital heart diseases who received prostaglandin E_1_ therapy from April 2000 to May 2020.

**Results:**

Necrotizing enterocolitis was observed in 10 patients (3.0%). Their average gestational age and birth weight were 38.2 weeks and 2783.5 g, respectively. The median age at diagnosis was 8.0 days (2–70 days). One patient was diagnosed with necrotizing enterocolitis stage IIA, five with stage IIB, two with stage IIIA, and two with stage IIIB; two (20%) received surgical treatment. The duct dependent pulmonary circulation group had higher frequencies of necrotizing enterocolitis (4.4%) than the duct dependent systemic circulation (2.0%) and parallel circulation (1.3%) groups. The necrotizing enterocolitis and the other groups had significantly different birth weight (2783.5 g vs 3170.9 g, respectively) and gestational age (38.2 weeks vs 39.1 weeks, respectively). Gestational age under 38 weeks (OR 8.87, *p* = 0.002), birth weight of < 2500 g (OR 5.1, *p* = 0.042), need for mechanical ventilation (OR 4.6, *p* = 0.021), parenteral nutrition (OR 107.7, *p* < 0.001), and functional single ventricle (OR 5.8, *p* = 0.009) were significant risk factors. The case-fatality rate was higher in the necrotizing enterocolitis (40.0%) than in the other group (8.3%, *p* = 0.009).

**Conclusions:**

Three percent of full-term infants with duct dependent congenital heart diseases developed necrotizing enterocolitis. Neonates with low birth weight, gestational age less than 38 weeks, functional single ventricle, or receiving assisted mechanical ventilation or parenteral nutrition are at increased risk.

## Introduction

Necrotizing enterocolitis (NEC) is a medical and/or surgical disease caused by necrosis of the intestinal mucosa or entire gastrointestinal layer of newborns. Although NEC typically occurs in preterm infants, approximately 10% of NEC cases have been reported in full-term infants [1]. In full-term infants, an association between congenital heart disease (CHD) and NEC has been described in the literature [2–4]. Unlike NEC in preterm infants, primary hypoxia/ischemia rather than inflammation has been suggested as a possible mechanism of NEC in full-term infants with CHD [5–8].

Duct dependent congenital heart disease (DD-CHD) is comprised of heart lesions that rely on patent arterial ducts to supply pulmonary or systemic blood flow [9]. Infants with DD-CHD may develop cyanosis/hypoxia or decreased perfusion because of the closure of their patent ductus arteriosus (PDA) within hours or days of birth. In patients with DD-CHD, administration of prostaglandin E1 (PGE1) helps maintain the medical palliative shunt until an interventional procedure can be performed [10]. However, the unique anatomy in patients with DD-CHD and decreased cardiac output such as systemic desaturation and decreased abdominal aortic blood flow may cause a decrease in mesenteric circulation, which may lead to NEC [5, 11–13].

Although several studies have reported that infants with DD-CHD are at a higher risk of developing NEC than infants with other CHD, data on the clinical characteristics and risk factors of NEC in the former are still scarce [13, 14]. This study aims to evaluate the clinical characteristics and risk factors of NEC in full-term infants with DD-CHD treated with PGE1.

## Methods

This is a retrospective study performed in the tertiary neonatal intensive care unit of the Samsung Medical Center (Seoul, Republic of Korea). Patient data from April 2000 to May 2020 were collected. This study was approved by the Institutional Review Board (approval number: SMC 2021–07–050-001). Informed consent was waived due to the retrospective study design. Full-term infants with DD-CHD in whom PGE1 was administered within 7days after birth were included in the study. Gestational age of ≥37 + 0 weeks was defined as full-term. Infants with DD-CHD were grouped into three categories: (i) duct dependent pulmonary circulation (e.g., pulmonary atresia and tetralogy of Fallot), (ii) duct dependent systemic circulation (e.g., hypoplastic left heart syndrome, coarctation of aorta, and interruption of the aortic arch), and (iii) duct dependent without an adequate mixing of blood between the two circulations (e.g., transposition of the great arteries) [15].

NEC was defined as stage IIA or higher according to the modified Bell’s staging. The stages were defined based on the clinical findings on feeding intolerance, abdominal distention, apnea, and radiographic findings, including the presence of pneumatosis intestinalis, portal venous gas, and/or pneumoperitoneum [1].

Data on demographic and clinical characteristics such as gestational age, birth weight, sex, and CHD type (including whether the patient had a functional single ventricle), age at NEC diagnosis, treatment of NEC (surgical or medical), feeding status at the time of NEC diagnosis, and whether the patients were mechanically ventilated were collected. When ventilator care was performed in the time between initial PGE1 administration and hemodynamic intervention (balloon valvuloplasty or shunt operation), the patients were defined as mechanically ventilated. The total administration time of PGE1 was noted. In the case of patients with NEC, the time from the beginning of PGE1 infusion to NEC diagnosis was noted. The average oxygen saturation (SpO2) of each patient 12 h before the end of PGE1 infusion was calculated. In patients with NEC, the average SpO2 12 h prior to NEC diagnosis was collected. Additionally, for patients with NEC, the mode of delivery and whether the patient’s mother had chorioamnionitis or pregnancy induced hypertension were collected.

### Statistical analysis

The data were analyzed using SPSS statistics 27 (IBM Corp. Armonk, New York, USA). The demographic data were presented as the number, percentage, and mean ± standard deviation (range) or median (interquartile range (IQR)). Categorical variables between the NEC and non-NEC groups were compared using the chi-square or Fisher’s exact test. Continuous variables were compared using the *t*-test or Mann–Whitney U test.

For risk factor analysis, the chi-square or Fisher’s exact test were conducted for categorical variables. Because early-term birth can be associated with adverse perinatal outcome, association of gestational age and occurrence of NEC were investigated between < 38 weeks of gestation and ≥ 38 weeks of gestation [16]. Also, as very low birth weight infants have been reported as an independent risk factor for NEC in patients with CHD, we investigated the association between low-birth-weight infants (LBWIs) and development of NEC in full-term DD-CHD patients with a cut-off birth weight of 2500 g (cut -off of Low-birth-weight infants) [17, 18]. Multivariate analysis was not performed due to the low number of events in this study. *P* values of <.05 were considered statistically significant.

## Results

A total of 335 infants (male: 208 (62.1%); female: 127 (37.9%)) with DD-CHD were identified during the study period. Their baseline characteristics are presented in Table 1. The median gestational age of the infants was 39.0 weeks (IQR 38.3–39.9 weeks, range: 37.0–42.1 weeks) and the average birth weight was 3159 ± 491.3 g (range: 1610–4550 g). The median administration time of PGE1 was median 159 h (IQR 103-238 h, range: 34–2467 h).

**Table 1:** Demographic and clinical characteristics of patients

	No NEC (***n*** = 325)	NEC (***n*** = 10)	***P***-value
**Gender, Male (%)**	203 (62.5)	5 (50.0)	0.513
**Mean birth weight, gram**	3170.9 ± 480.4	2783.5 ± 696.8	0.014^†^
**Birth weight, gram (%)**			
≥ 2500 (ref)	300 (92.3)	7 (70)	
< 2500	25 (7.7)	3 (30)	0.008
**Median gestational age, weeks (IQR)**	39.1 (38.3–40.0)	37.9 (37.3–39.4)	0.011^‡^
**Median PGE**_**1**_**infusion, hours (IQR)**	159.0 (104–237)	437 (328–940)	0.632^‡^
**Average oxygen saturation, percent**	87.6 ± 7.2	89.1 ± 7.8	0.399^†^
**Feeding status (%)**			
Full enteral feeding (ref)	323 (99.4)	6 (60)	
Parenteral nutrition or NPO	2 (0.6)	4 (40)	< 0.001
**Ventilator care (%)**			
No (ref)	245 (75.4)	4 (40)	
Yes	80 (24.6)	6 (60)	0.021
**Functional single ventricle (%)**			
No (ref)	258 (79.4)	4 (40)	
Yes	67 (20.6)	6 (60)	0.009

*NEC* necrotizing enterocolitis, *IQR* interquartile range, *ref* reference group, *NPO* nil per os, *PGE*_*1*_ prostaglandin E_1_

^†^ Student’s *t*-test

^‡^ Mann–Whitney U test

NEC was observed in 10 infants (3.0%) during the study period (Table 2). Of these, one (10%) was diagnosed with NEC stage IIA, five (50%) with stage IIB, two (20%) were stage IIIA, and two (20%) with stage IIIB. The latter two infants with stage IIIB NEC (20%) received surgical therapy, while the remaining eight (80%) received only medical therapy. Considering the surgical findings in patients with NEC IIIB, one had color change in descending and sigmoid colon, and another had necrosis in ascending to descending colon. Histopathologic findings from these two patients were consistent with NEC. At the time of NEC diagnosis, six of these infants (60%) had received full enteral nutrition, two (20%) received combined parenteral and enteral nutrition, and the other two (20%) had received total parenteral nutrition. The median gestational age of these 10 patients was 37.9 weeks (IQR 37.3–39.4, range: 37.0–39.7 weeks), the average birth weight was 2783.5 g ± 696.8 (range: 1610–3900 g), the median duration for PGE1 infusion was median 437 h (IQR 328–940, range: 44–1411 h), and the median age at NEC diagnosis was 8.0 days (IQR 4.5–20.5, range: 2–70 days). Out of the ten patients with NEC, four patients were available for histopathologic findings of maternal placenta. Additionally, there was no evidence of chorioamnionitis in any of the four patients.

**Table 2:** Patient and clinical information of NEC

Case	Sex	Birth weight (gram)	Gestational Age (weeks)	Type of CHD	FSV	Age at NEC diagnosis (days)	Feeding status at NEC diagnosis	Mode of delivery	Pathologic chorioamnionitis/Maternal PIH	NEC Bell’s stage	Surgical treatment of NEC	Mortality
1	M	3130	37 + 5	PA	Yes	2	T	VD	NA/No	IIIA	No	No
2	M	3150	38 + 3	PA	Yes	3	P	CS	NA/NA	IIB	No	No
3	F	1610	37 + 1	CoA	No	5	F	CS	No/No	IIB	No	No
4	F	1990	37 + 3	HLHS	Yes	70	F	VD	No/No	IIIA	No	Yes
5	F	2110	37 + 6	TOF with PA	No	37	F	CS	No/No	IIB	No	No
6	M	3015	39 + 2	PA	Yes	6	F	VD	NA/NA	IIIB	Yes	No
7	M	2617	37 + 6	Ebstein’s anomaly	Yes	11	F	VD	No/No	IIB	No	No
8	M	2900	39 + 5	TGA	No	5	P	VD	NA/essential hypertension	IIA	No	Yes
9	F	3313	39 + 4	PA with VSD	No	10	T	NA	NA/NA	IIB	No	Yes
10	F	3000	37	PA IVS	Yes	15	F	VD	NA/NA	IIIB	Yes	Yes

*NEC* necrotizing enterocolitis, *CHD* congenital heart disease, *FSV* functional single ventricle, *PIH* pregnancy induced hypertension, *PA* pulmonary atresia, *CoA* coarctation of aorta, *HLHS* hypoplastic left heart syndrome, *TOF* tetralogy of Fallot, *TGA* transposition of great arteries, *VSD* ventricular septal defect, *IVS* intact ventricular septum, *T* total parenteral nutrition, *P* partial enteral feeding, *F* full enteral feeding, *VD* vaginal delivery, *CS* cesarean section, *NA* not available

The frequencies of NEC in infants with DD-CHD were calculated based on the type of duct dependent lesion (Table 3). In infants with duct dependent pulmonary circulation, 4.4% (7/158) had NEC. In duct dependent systemic circulation, 2.0% (2/101) had NEC. In infants with inadequate mixing of systemic and pulmonary circulations (parallel circulation), 1.3% (1/76) had NEC. A total 80 infants (24.6%, 80/325) in the non-NEC group and 6 (60%, 6/10) in the NEC group were mechanically ventilated. The infants of the NEC and non-NEC groups have some significantly different characteristics (Table 1). The mean birth weight of the infants in the NEC group was significantly lower (2783.5 g) than that of the infants in the non-NEC group (3170.9 g, *p* = 0.014). There was also a significant difference in the mean gestational age (38.2 weeks (NEC) vs 39.1 weeks (non-NEC), *p* = 0.011). However, the gender, DD-CHD type, PGE1 infusion time, and SpO2 were not significantly different.

**Table 3:** Type of ductal-dependent congenital heart disease

Type of DD-CHD	No NEC (%) (***n*** = 345)	NEC (%) (***n*** = 10)	***P***-value
DD-pulmonary circulation (ref)	151 (95.6)	7 (4.4)	
DD-systemic circulation	99 (98.0)	2 (2.0)	0.489
Parallel circulation	75 (98.7)	1 (1.3)	0.443

*DD-CHD* ductal-dependent congenital heart disease, *ref* reference group

The risk factor analysis showed that infants born under 38 weeks of gestation were at a higher risk of developing NEC with an odds ratio (OR) of 8.87 (95% confidence interval (CI) 2.4–32.6, *p* = 0.002) (Table 4). Low birth weight was significant risk factor with an OR of 5.1 (95% CI 1.3–21.1, *p* = 0.042). Infants who received partial or total parenteral nutrition had a higher risk of developing NEC than those who received full enteral nutrition (OR 107.7, 95% CI 16.4–705.4, *p* < 0.001). Patients who were mechanically ventilated were also at a greater risk of developing NEC (OR 4.6, 95% CI 1.3–16.9, *p* = 0.021). In addition, patients with functional single ventricle had higher risk for NEC (OR 5.8, 95% CI 1.6–21.1, *p* = 0.009).

**Table 4:** Risk factors for NEC

Variables	Univariate analysis		
	OR	95% CI	*P*-value
Sex, male	0.60	0.17–2.12	0.513
Gestational age			
38–42 weeks (ref)			
37–37 + 6 weeks	8.87	2.41–32.63	0.002
PGE_1_ infusion time			
< 1 week (ref)			
1–2 weeks	0.61	0.12–3.06	0.717
2–4 weeks	1.10	0.13–9.44	1.000
≥ 4 weeks	2.56	0.28–22.97	0.375
Birth weight			
≥ 2500 g (ref)			
< 2500 g	5.14	1.25–21.12	0.042
Nutrition			
Full enteral (ref)			
Partial or NPO	107.67	16.43–705.38	< 0.001
Type of DD-CHD			
DD-pulmonary (ref)			
DD-systemic	0.44	0.09–2.14	0.489
Parallel	0.29	0.04–2.38	0.443
Functional single ventricle	5.78	1.59–21.05	0.009
Ventilator care	4.59	1.26–16.69	0.021

*NEC* necrotizing enterocolitis, *OR* odds ratio, *CI* confidence interval, *ref* reference group, *PGE*_*1*_ prostaglandin E_1_, *NPO* nil per os, *DD-CHD* ductal-dependent congenital heart disease, *DD* ductal-dependent

In the outcome analysis, the case-fatality rate of infants in the non-NEC group was 8.3% (27/325), while at 40% (4/10), it was significantly higher in infants in the NEC group (*p* = 0.009).

## Discussion

We investigated the frequency of NEC in patients with DD-CHD receiving PGE1. We noted a 3.0% frequency of NEC (10/335). The median age at NEC diagnosis was 8.0 days. The NEC and non-NEC groups had significantly different birth weights and gestational ages. In addition, univariate analysis showed that patients who were born under 38 weeks of gestation, had a birth weight < 2500 g, were on parenteral nutrition or ventilator care, or had single ventricular heart disease were at a higher risk of developing NEC. In terms of patient outcome, the case-fatality rate was significantly higher in the NEC group.

For full-term infants, the incidence of NEC was reported to be 0.05–0.71 per 1000 live births [3, 19, 20]. The frequency of NEC in patients with CHD has been reported to be 2.9–3.7% in previous literature [13, 21, 22]. A more variable frequency of 0.3–5% has been reported for patients with DD-CHD [11, 13, 23]. In the present study, 3.0% of NEC was reported in full-term infants with DD-CHD. This difference in the frequency of NEC may be attributed to different inclusion criteria, variable feeding regimens, and different severity of the patients among the studies [3, 11, 13, 19–23].

Some previous studies have reported earlier presentation of NEC in full-term infants or in infants with CHD and a median postnatal age of 4 days [8, 24]. However, there are conflicting data on whether the average age of full-term and preterm infants at NEC onset is significantly different (28.2 vs 19.6 days) [25]. In patients with DD-CHD, a median age of 16–24 days at NEC onset has been reported including preterm infants [11, 13]. Unlike previous studies, we considered only full-term infants and reported that the median age at the time of NEC diagnosis was 8.0 days. In addition, 50% of the infants developed NEC within 7 days after birth (Table 2). Mesenteric circulatory insufficiency due to systemic desaturation and decreased cardiac output influences the onset of NEC [5, 7, 11, 12]. Poor systemic perfusion is caused by systemic-to-pulmonary arterial shunt through PDA, which is believed to cause ischemic injury of the intestine [8, 26]. The ischemic injury may be responsible for the earlier onset of NEC in full-term DD-CHD infants. As the three types of DD-CHD show different hemodynamics, we initially assumed that NEC frequencies will also be different depending on the DD-CHD type. In this study, NEC was more prevalent in patients with duct dependent pulmonary circulation (Table 3).

We conducted a risk factor analysis and observed that gestational age < 38 weeks, birth weight of < 2500 g, parenteral nutrition, ventilator care, and single ventricular heart disease are significant risk factors for NEC in patients with DD-CHD.

In general, it is well known that infants with lower gestational age had higher risk of developing NEC [27]. Early-term infants are known to be associated with increased adverse outcomes such as need for respiratory support, hypoglycemia, feeding intolerance, or needing care from a neonatal intensive care unit [16, 28]. Although to what extent is unclear, our findings show relative immaturity also has an influence on developing NEC in early-term infants with DD-CHD. However, future studies with larger sample sizes are needed to confirm this finding.

There were three patients with NEC who were under 2500 g in this study. Out of these three patients, two infants with birth weights of 1990 and 2110 g were diagnosed with NEC at a postnatal age of over 30 days (patient 4 and 5 in Table 2). A relatively longer exposure to duct dependent hemodynamics before therapeutic intervention might put these infants at a higher risk of developing NEC.

There was an article describing increased risk of NEC in DD-CHD patients with single ventricle physiology with an OR of 2.82 [11]. Our finding is compatible with this finding, and the relatively higher chance of decreased mesenteric perfusion in patients with single ventricular heart disease is thought to be related to this result.

Generally, enteral nutrition helps in the maturation of the immune system of infants. It also helps in reducing NEC risk by preventing infection through the central line, which might occur with prolonged parenteral nutrition [29, 30]. However, patients with DD-CHD on enteral nutrition show a higher incidence of NEC than those without enteral nutrition in a previous study (though statistically insignificant) [11]. A recent study has reported that human milk diets reduced the risk of NEC in complex CHD patients [31]. We assumed that enteral feeding will not affect incidence of NEC in full-term infants with DD-CHD, because pathophysiology in this population was believed to be related more to hemodynamics rather than to inflammation or gut maturation. This assumption was compatible with our current finding that infants receiving partial parenteral or total parenteral nutrition had higher incidence of developing NEC. However, this finding may only reflect that those patients had significant systemic illnesses or hemodynamic instability preceding feeding intolerance. In addition, as we did not collect data on the exact feeding volume and the type of milk used, we could not investigate the associations between feeding volume, human milk diet, and NEC. In the same way, association between need for mechanical ventilation and development of NEC is also thought to be more affected by the patient’s systemic illness or hemodynamic instability.

The overall mortality among neonates with NEC has been reported to be 20–40% [17]. Previous studies have reported case-fatality rates of 24–47% in patients with DD-CHD and NEC [11, 13]. In the present study, case-fatality rates of 40% among patients with NEC has been reported, which is comparable to that of previous studies. Hence, careful monitoring is needed because of a high case-fatality rate and the possibility of morbidity in such infants.

This study has several limitations as well. It is a single-center study with a limited number of cases. In addition, our analysis may be underpowered because of the low event rate and small sample size. Hence, future studies with larger sample sizes should be conducted. Moreover, because we retrospectively reviewed the data to identify patients with DD-CHD and NEC by searching a compatible ICD-10 code, there is a possibility that some patients were missed.

In DD-CHD patients, severe NEC may cause significant delay in timely hemodynamic intervention, and NEC itself can be devastating for patients. To prevent NEC, making efforts to maintain sufficient gut perfusion will always be necessary in this patient group. Also, rigorous monitoring of early signs of NEC is important, as early detection and proper intervention are crucial to improve the outcomes of NEC. The results of our study may help clinicians distinguish at-risk populations in full-term DD-CHD patients who require special attention.

## Conclusion

Three percent of full-term infants with DD-CHD developed NEC. Neonates with low birth weight, gestational age less than 38 weeks, functional single ventricle, or receiving assisted mechanical ventilation or parenteral nutrition are at increased risk.

## Data Availability

The datasets used and/or analyzed during the current study are available from the corresponding author on reasonable request.

## Abbreviations

NEC: Necrotizing enterocolitis
CHD: Congenital heart disease
DD-CHD: Duct dependent congenital heart disease
PDA: Patent ductus arteriosus
PGE1: Prostaglandin E1
LBWI: Low birth weight infant
